# Biomechanical Analysis of Micromotion of Proximal Interphalangeal Joint Arthrodeses During Activities of Daily Life In Vitro

**DOI:** 10.3390/jcm14134420

**Published:** 2025-06-21

**Authors:** Michael Millrose, Till Ittermann, Hans Christoph Vonderlind, Maximilian Willauschus, Johannes Rüther, Hermann-Josef Bail, Markus Geßlein

**Affiliations:** 1 Department of Trauma Surgery and Sports Medicine, Garmisch-Partenkirchen Medical Centre, 82467 Garmisch-Partenkirchen, Germany; 2 Department of Orthopedics and Traumatology, Paracelsus Medical University, 90419 Nuremberg, Germany; maximilian.willauschus@klinikum-nuernberg.de (M.W.); johannes.ruether@klinikum-nuernberg.de (J.R.); hermann-josef.bail@klinikum-nuernberg.de (H.-J.B.); markus.gesslein@klinikum-nuernberg.de (M.G.); 3 Institute for Community Medicine, SHIP/Clinical-Epidemiological Research, University of Greifswald, 17489 Greifswald, Germany; till.ittermann@uni-greifswald.de; 4 Department of Trauma Surgery, HELIOS Hospital Schwerin, University Campus of MSH Medical School Hamburg, 19055 Schwerin, Germany; h.c.vonderlind@gmail.com

**Keywords:** PIJ arthrodesis, interfragmentary strain arthrodesis, primary stability arthrodesis, early active movement

## Abstract

**Background/Objectives:** Proximal interphalangeal joint (PIJ) arthrodesis is a common surgical intervention for patients with PIJ osteoarthritis or trauma-related joint destruction. The objective of this study was to evaluate the biomechanical stability of various arthrodesis techniques under forces comparable to activities of daily living (ADL) to assess their suitability for early active movement protocols. **Methods:** In this in vitro study, composite cylinders simulating PIJ arthrodesis were subjected to standardized fusion angles of 40° using different fixation techniques, including crossed K-wires, compression screws, cerclage wires, tension band wiring, anatomical fixation plates, and locking grid plates. Forces representing ADLs such as typing, holding a pencil, carrying weight, and opening a jar were applied using a universal testing machine in a four-point bending setup. Micromotion and gap clearance were calculated and analyzed. **Results:** Techniques involving compression, such as compression screws, tension bands, and cerclage wires, exhibited lower micromotion and gap clearance under forces up to 17 N, suggesting potential suitability for early active movement protocols. In contrast, fixation plates demonstrated structural failure or excessive clearance during early active motion ADLs. K-wires showed intermediate results with moderate gap clearance and micromotion. **Conclusions:** Compression-based fixation techniques for PIJ arthrodesis may permit early active movement without external stabilization, while fixation plates are prone to failure under ADL forces. Further dynamic biomechanical testing and clinical studies are recommended to confirm these findings.

## 1. Introduction

Destruction of the proximal interphalangeal joint (PIJ) of the finger, whether it is derived primarily or secondarily due to trauma or rheumatoid diseases, frequently results in the deterioration of the range of motion and pain during activities of daily living. This may result in a decline in global hand function [1]. PIJ osteoarthritis represents the second most prevalent cause of pain and is among the most common forms of osteoarthritis in the hand [2].

Fusion of the PIJ will result in stable arthrodesis, which allows a strong and painless grip [3]. This is of particular relevance to individuals engaged in manual labor, such as artisans. However, it should be noted that nearly all activities of daily living may benefit, with the greatest impact being observed in cases where the dominant hand is affected. As most of the described fusion techniques in the literature—for example, using K-wires, tension-band, or compression screws—require a certain amount of immobilization to achieve bony fusion in a reasonable time, there is concern that adjacent fingers and joints (e.g., metacarpophalangeal or distal interphalangeal joints) might suffer in terms of mobility. Additionally, the entire hand or upper extremity may experience a decline in dexterity. Therefore, in the context of hand surgery, the promotion of early active movement, particularly in activities of daily living, is of paramount importance [4]. This approach has been demonstrated not only to enhance outcomes but also to promote greater autonomy for the patient undergoing surgery.

In previous studies, the force exerted on the PIJ during activities of daily living (ADLs) has been examined. Keyboard typing exerts a force of 7 newtons (N) on the joint, whereas gripping a pen-like object, especially with the index finger, might result in a force of up to 17 N. These activities and their resulting forces can be assigned to an early active motion protocol. In contrast, activities like carrying weights or opening a jar will exert forces of up to 84–164 N and are therefore not recommended [5,6]. The exemplary fusion technique of intraosseous wiring has been demonstrated to result in failure in biomechanical studies [7].

Two distinct categories of fracture or arthrodesis healing have been identified: primary or direct healing, characterized by absolute stability achieved through compressive preload and friction, and secondary or indirect healing, marked by the occurrence of micro movements [8]. Fixation with absolute stability signifies that there is no motion of the fusion partners under physiological load. The healing process is characterized by the process of remodeling. The degree of micromotion exhibited by the implant in question, whether it be K-wires or compression screws, is subject to variation. In cases where interfragmentary strain is excessive, leading to unstable fixation, the potential for bony bridging and fusion of the arthrodesis gap may be compromised, resulting in non-union and implant failure over time. In biomechanical studies of trauma surgery, it has been demonstrated that smaller interfragmentary gaps with reduced micromotion can facilitate optimal healing [9,10].

To our knowledge there are no biomechanical studies investigating the clearance of the fusion gap during ADLs. The objective of this biomechanical study is to evaluate the micromotion of the arthrodesis gap with different implants at forces comparable to early active motion ADLs. The stability is evaluated and classified according to the probability of bony healing in response to gap widening during strain. Additionally, the impact of employing a technique that involves compressing the arthrodesis gap on micromotion is examined.

## 2. Materials and Methods

The experimental setup and measurement system were comparable to those reported in previous studies [11]. The following concise synopsis will be provided.

The specimens, which were designed to simulate the fused PIJ, consisted of 42 mm-long phalangeal equivalents. They were fabricated from short fiber-reinforced epoxy cylinders with a diameter of 10 mm and a thickness of the corticalis of 1.5 mm (fourth generation, Sawbone Europe AB, Malmö, Sweden). To simulate cancellous bone within the corticalis, composite cylinders were filled with cellular rigid polyurethane foam (strength 15).

Each fusion was stabilized at a standard angle of 40°, representing a compromise between the advocated PIJ arthrodesis angles of 20–50°, depending on the finger [12].

The arthrodesis techniques evaluated included crossed K-wires, crossed compression wires, cerclage with anti-rotation wire, tension band wiring, compression screws, anatomical fixation grid plates, and locking grid plates.

The steel K-wires utilized in this study included l with a diameter of 1.2 mm. The compression wire utilized in this study featured a threadless section measuring 10 mm in length (Koenigsee, Allendorf, Germany). To achieve compression of arthrodesis, three turns of the trailing thread were advanced into the distal cortical bone. The compression wire’s placement enabled positioning of the tip, with the wire’s threads and main body following until compression was applied to the fusion site. The wire-based arthrodesis techniques crossed the fusion site at a 45° angle in the lateral plane and were placed 8 mm proximally and distally to the arthrodesis site.

The compression screw (2.2 mm diameter, 26 mm length; Medartis, Basel, Switzerland) was implanted from proximal to distal, perpendicular to the fusion site, to generate compression. The screw was positioned in a manner that ensured its complete burial within the cylinders. The screw exhibited two threads with different pitches and a 10 mm threadless section, ensuring secure cortical fixation and effective compression of arthrodesis. The diameter of the compression screw ranged from 1 mm at the object’s tip to 1.8 mm at the second thread area.

The cerclage with anti-rotation wire as well as the tension band wiring were performed using a 0.8 mm cerclage wire and a 1.2 mm K-wire, in accordance with well-established techniques [13,14].

Anatomical and locking grid plate fixation techniques both employed a 0.8 mm low-profile plate (4 × 2 grid fixation plate), which was meticulously contoured to conform to the cylinder and the targeted angle of 40°. To ensure adequate fixation on each side of the fusion, eight cortices were stabilized using 1.5 mm cortical or locking screws (Medartis, Basel, Switzerland).

The biomechanical experiments were conducted with a universal testing machine (Zwick Z050^®^, Zwick GmbH & Co. KG, Ulm, Germany) in a four-point bending setup. The force application arms were applied 3 mm proximally and distally from the arthrodesis site onto the composite cylinders. The proximal and distal ends of each specimen were embedded in acrylic cylinders. The utilization of cylindrical supports was imperative to facilitate compensatory rotational movement and ensure the maintenance of a consistent contact area during the loading process.

Subsequent to the fusion of the composite cylinders employing the aforementioned techniques, force was applied at a rate of 100 mm per minute in 0.5° increments until reaching 10° of flexion or extension. The requisite force was documented in real-time using testXpert III 1.1 software (Zwick GmbH & Co. KG, Ulm, Germany). A failure was defined if the resulting fusion angle, determined by the iteration, would turn negative.

The calculated normal forces acting on the PIJ for the ADLs—namely, typing, playing piano, holding a pencil, carrying a bag, and opening a jar—were oriented into an extension force for the fused joint [5]. To ensure comprehensiveness, the alterations in the fusion angle of the PIJ during flexion were likewise computed.

The association between the force applied on the PIP joint and the fusion angle was determined using median regression models for each technique individually. The potential non-linear associations were modeled using fractional polynomials [15]. To test for differences in the method regarding the association of the force with the angle, we tested interaction terms between the force and method on the angle in the regression models. Here, the K-wire was considered a reference method, and all other methods were compared to it separately. The extension calculation was performed using the following models: 7 N (keyboard typing), 19.3 N (piano playing), 17 N (pencil writing), 84 N (carrying a normal weight), and 164.4 N (jar opening).

## 3. Results

The following calculated forces from the literature were used for typical ADLs—specifically, the forces required for keyboard typing, piano playing, writing with a pencil, carrying a normal weight, and opening a jar—were determined to be 7 N, 19.3 N, 17 N, 84 N, and 164.4 N, respectively [5]. These forces act in the extension direction on the PIJ.

**Figure 1:**
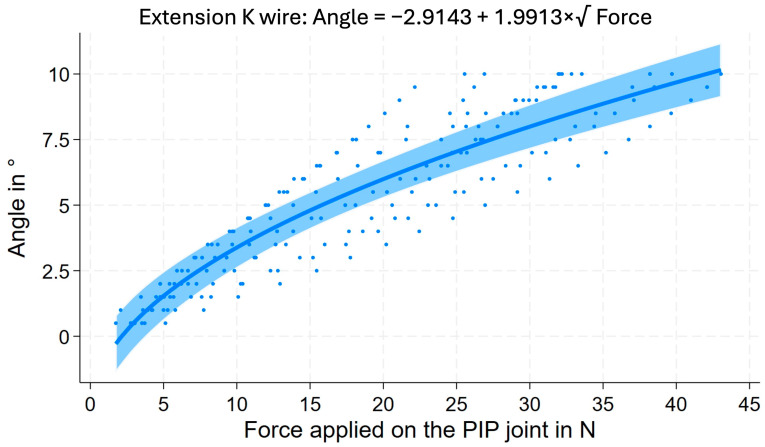
Iteration graph and the underlying mathematical equation for the relationship of force applied on the PIJ with resulting change of the fusion angle with force applied in extension for K-wire (n = 10).

The iteration of the relationship of applied force resulting in a change of the fusion angle was computed based on the measurements (n = 10 for each technique) of forces necessary to bend the fusion angle from 40° by ten degrees (in 0.5° steps) in extension and, respectively, flexion (Figure 1). The equations for each technique are located in the Appendix A (see Table A1).

The resulting alterations in fusion angulation and the subsequent clearance of the gap in the middle and the palmar site of the PIJ with forces acting in the extension direction are illustrated in Table 1.

The forces exerted on the PIJ during early active movement, typing, penciling, and piano playing result in a maximum opening of the fusion of 0.31 mm to 0.51 mm when applying techniques that involve compression force. Dorsal plate fixation has been demonstrated to result in failure for the fixation plate, with a clearing of 1.45 mm for the locking plate. The K-wires, utilized as a standard of reference, exhibited a maximum opening of 0.51 mm.

**Figure 2:**
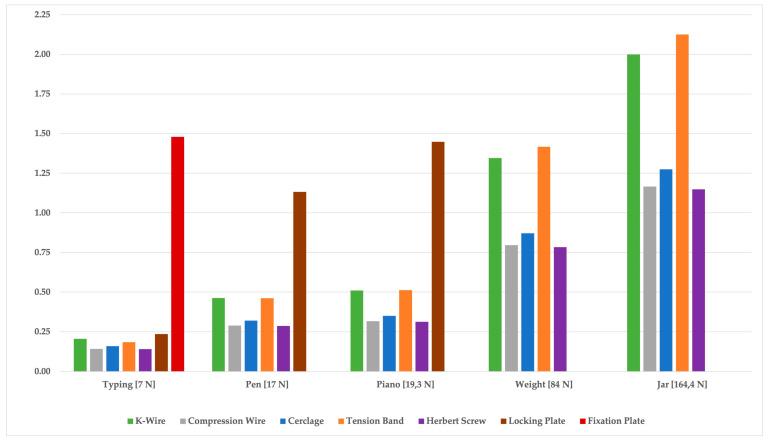
Comparison of the palmar clearance of arthrodesis during ADLs acting in extension with different techniques.

The application of forceful ADLs, assuming a normal weight and jar opening, yielded clearances of up to 2.13 mm, resulting in the failure of both plates (Figure 2).

## 4. Discussion

This biomechanical study of PIJ arthrodeses demonstrated that micromotion occur during activities of daily living. Early active movement activities, such as penciling or typing, result in clearance gaps of less than 1 mm for all fusion techniques, with the exception of plating, which demonstrates failure. From a biomechanical perspective, it can be hypothesized that a fusion of arthrodesis should occur in a reasonable timeframe with these small movements, without the need for external stabilization.

A systematic review by Millrose et al. from 2022 [3] demonstrated that all fusion techniques result in an arthrodesis of the PIJ within a reasonable time frame, with an average non-union rate of 6.3%. Despite the absence of statistically significant evidence in the extant published literature, there appears to be an emerging trend toward techniques involving compression of the fusion gap [3]. It is reasonable to hypothesize that the application of compression will result in a reduction of micromotion in arthrodesis, attributable to the frictional forces between the bony components.

A clinical study by Leibovic and Strickland compared the fusion rates of PIJ arthrodeses with different techniques. They could show a high rate of non-unions with K-wires (21%) and only a few with compression screws (0%) as well as tension bands (5%). Even though they included only four arthrodeses with plate fixation, they reported a radiographic non-union rate of 50% [16]. The duration of immobilization was not reported. These clinical results are in line with our biomechanical study showing an increased non-union rate with excessive clearing of the gap for techniques without compression.

Another clinical study by Stahl and Rozen from 2001 [17] analyzed the results of 41 tension band arthrodeses of the PIJ. They reported their immobilization period with 4–6 days. They reported no non-unions [17]. This is in concurrence with the results of this study, implying, from a biomechanical viewpoint, that tension-band wiring has the potential of fusing the PIJ in early active motion.

A substantial body of the extant literature on the subject reports the application of some form of postoperative immobilization in arthrodesis of the PIJ. The duration was found to be an average of four to six weeks [3]. Immobilization can result in a reduction of the range of motion of adjacent joints or fingers, which necessitates occupational therapy during and after the immobilization period [18]. The fusion will result in a modified global hand function due to the coupling of adjacent fingers, as evidenced by the quadriga effect. The potential for avoiding additional impairments through prolonged external stabilization of the hand is a salient consideration [19].

Consequently, the potential for early active movement during ADLs without external stabilization would be advantageous. It is imperative that there is adequate primary stability, which is comprised of the stability provided by the implant and the interdigitation of the two fusion partners. This primary stability can be enhanced by applying compression. In the event that the primary stability results in an excessive amount of micromotion of the fusion gap, the implementation of an external stabilization, such as a splint, becomes imperative.

As demonstrated by Claes et al. in a series of biomechanical studies examining the mechanobiology of bone healing, the presence of a certain degree of micromotion is indispensable for stimulating bone healing. The interfragmentary strain hypothesis was first proposed by Perren and Cordey in 1980 [20]. The hypothesis posits that if the interfragmentary strain (IFS) is elevated, defined as the interfragmentary micromotion divided by the length of the gap, a non-union would ensue [21]. The interfragmentary strain in this biomechanical study for the techniques not failing ranged from 1.4% to 5.1% for early active movement ADLs. The absence of compression in the techniques demonstrated a 150% increase in interfragmentary strain in comparison to the techniques that employed compression (5.1% IFS K-wire vs. 2.9% IFS compression screw with ADL penciling). From a biomechanical perspective, all fusion techniques for the PIJ, with the exception of plating, should be deemed suitable for an early active motion approach on ADLs.

The efficacy of both plate fixations is demonstrated to be inadequate, failing even in low-power ADLs with a force of up to 17 N. The forces acting on the PIJ during object-grasping activities are oriented in the extension of arthrodesis [5]. The plates, made of titanium, utilized in this biomechanical study possess a thickness of 0.6 mm, which serves as the sole impediment to the applied forces. The failure of plate fixation appears to be primarily due to plastic deformation of the implant rather than loosening. This finding indicates that the construct exhibited inadequate structural rigidity to adequately resist the mechanical stresses exerted on the fusion site. Plates that are too thin, narrow, or improperly contoured may be susceptible to bending under loading [22]. In the context of arthrodesis of the metacarpophalangeal joint of the thumb, the utilization of a 1 mm thick plate is customary, a choice that has the potential to induce alterations in behavior. Plates are generally affixed to the dorsal aspect of the PIJ during fusions, thus not on the tension side of the arthrodesis region [23]. Fixing the plate on the non-tension side may have further reduced its ability to resist bending forces. Plating on the tension side is known to better counter tensile forces, while fixation on the compression side can result in micromotion and eventual implant deformation [23,24]. It is imperative that arthrodesis of the PIJ using plate fixation be externally stabilized during ADLs. In the context of an early active movement protocol that involves mobilizing the fingers without the application of external forces, the plates are affixed to the tension side, thereby withstanding forces that are of a very high magnitude, which could be a compromise for using them and minimizing the impact on the ROM.

There are other factors influencing the decision for a certain technique of PIJ fusion. Plates or compression screws are significantly more expensive than K-wires or cerclage wires. Especially in health care systems that do not reimburse the implant used, this could be an influence. Also, the use of a certain technique often emerges due to individual surgeon preferences, their unique expertise, and comfort levels with specific implants [25,26]. Also, patient-specific anatomy might influence the use of a certain implant. Even though the dimensions of the proximal and middle phalanx are wider than the ones forming the distal interphalangeal joint (DIJ), they might be too small to fit a headless compression screw, especially on the little finger of a female. This is a known problem for arthrodesis of the DIJ [27].

This biomechanical study is subject to several limitations. First, the testing is of a static nature rather than dynamic. The potential failure of the implants during the period of bony consolidation of arthrodesis can be attributed to repetitive strain incurred during ADLs. It is hypothesized that implants with a higher diameter, such as the compression screw, would exhibit greater resistance to the applied strain when compared to smaller implants. However, given the rigidity of the former, there is a potential increased risk of cutting out. A biomechanical stability analysis, incorporating repetitive force application, should be incorporated to further refine the research design. Additionally, the scope of this biomechanical study has been confined to artificial bone specimens, with a defined fusion angle, thus limiting its generalizability. It is conceivable that the implant’s primary stability may vary depending on the angle of implantation. Consequently, the optimal fusion angle for a particular implant may be contingent on the implant’s design characteristics. Also, the biomechanical findings of this study were solely assessed on artificial bone. Even though epoxy cylinders are designed to biomechanically mimic human bone, it could be expected that human cadaveric bones would have different implant–bone interface behavior, depending on the quality of the bone, i.e., whether it is influenced by osteoporosis. Further biomechanical studies using human specimens, focusing on techniques able to withstand early active movement, should be done. Also, only primary stability can be tested by biomechanical studies; the healing biology of arthrodesis cannot be simulated. Additionally, the findings of this study are exclusively derived from biomechanical testing, thereby underscoring the potential for diverse fusion techniques in subsequent clinical investigations. These investigations aim to ascertain the feasibility of circumventing external fixation during early active movement activities within daily living contexts.

## 5. Conclusions

This is the first biomechanical study analyzing clearance of the fusion gap, an important aspect within IFS theory on the union of arthrodesis, and demonstrates that fusion techniques of PIJ arthrodesis, employing compression mechanisms such as compression screws, tension band, and cerclage, exhibit resilience to forces during early active motion ADLs. This finding suggests the potential for successful fusion. In the context of these ADLs, plate fixation has been demonstrated to be ineffective, necessitating the use of external stabilization mechanisms. The excessive clearing of arthrodesis in relation to the small diameter of the fusion partners might be one of the explanations for a higher rate of non-unions in techniques resulting in high IFS levels.

It is imperative that the results of this study be subjected to biomechanical testing in order to eliminate the possibility of implant failure during consolidation by repetitive strain. Such dynamic studies, as well as further investigations on the influence of using human specimens, may alter the findings of this study, focusing on primary stability. Furthermore, it is essential that clinical studies be conducted in order to verify the findings of this study.

## Figures and Tables

**Table 1:** Clearances of arthrodeses at the center and at palmar cortex during different ADLs acting in extension with different techniques; X = technique failed. Data are given as median ± 95% confidence interval, * *p* < 0.001.

Technique	ADL	Change of Fusion Angle	Clearance Center	Clearance Palmar
		[°]	[mm]	[mm]
Cerclage	Typing	1.83 (1.2–2.5) *	0.08 (0.05–0.11)	0.16 (0.10–0.22)
Cerclage	Piano	4.01 (3.6–4.4) *	0.17 (0.16–0.19)	0.35 (0.31–0.38)
Cerclage	Pencil	3.67 (3.2–4.1) *	0.16 (0.14–0.18)	0.32 (0.28–0.36)
Cerclage	Weight	9.96 (9.1–10.8) *	0.44 (0.40–0.47)	0.87 (0.80–0.95)
Cerclage	Jar	14.52 (13.0–16.0) *	0.64 (0.57–0.70)	1.27 (1.14–1.41)
Compression wire	Typing	1.61 (1.2–2.1) *	0.07 (0.05–0.09)	0.14 (0.10–0.18)
Compression wire	Piano	3.62 (3.3–3.9) *	0.16 (0.14–0.17)	0.32 (0.29–0.34)
Compression wire	Pencil	3.31 (3.0–3.6) *	0.14 (0.13–0.16)	0.29 (0.26–0.31)
Compression wire	Weight	9.10 (8.6–9.6) *	0.40 (0.38–0.42)	0.80 (0.75–0.84)
Compression wire	Jar	13.30 (12.4–14.2) *	0.58 (0.54–0.62)	1.17 (1.09–1.25)
Fixation Plate	Typing	16.83 (16.2–17.5) *	0.74 (0.71–0.77)	1.48 (1.42–1.54)
Fixation Plate	Piano	X	X	X
Fixation Plate	Pencil	X	X	X
Fixation Plate	Weight	X	X	X
Fixation Plate	Jar	X	X	X
Herbert Screw	Typing	1.61 (1.1–2.1) *	0.07 (0.05–0.09)	0.14 (0.10–0.18)
Herbert Screw	Piano	3.58 (3.2–3.9) *	0.16 (0.14–0.17)	0.31 (0.28–0.34)
Herbert Screw	Pencil	3.27 (2.9–3.6) *	0.14 (0.13–01.6)	0.29 (0.25–0.31)
Herbert Screw	Weight	8.97 (8.5–9.5) *	0.39 (0.37–0.42)	0.78 (0.74–0.83)
Herbert Screw	Jar	13.10 (12.2–14.0) *	0.57 (0.53–0.61)	1.15 (1.07–1.23)
K-Wire	Typing	2.35 (2.0–2.7)	0.10 (0.09–0.12)	0.21 (0.17–0.24)
K-Wire	Piano	5.83 (5.6–6.1)	0.25 (0.24–0.27)	0.51 (0.49–0.53)
K-Wire	Pencil	5.30 (5.0–5.5)	0.23 (0.22–0.24)	0.46 (0.44–0.48)
K-Wire	Weight	15.34 (14.3–16.3)	0.67 (0.63–0.72)	1.35 (1.25–1.43)
K-Wire	Jar	22.62 (20.9–24.3)	1.00 (0.92–1.08)	2.00 (1.84–2.15)
Locking Plate	Typing	2.70 (2.5–2.9) *	0.12 (0.11–0.13)	0.24 (0.22–0.25)
Locking Plate	Piano	16.48 (16.0–17.0) *	0.72 (0.70–0.75)	1.45 (1.41–1.49)
Locking Plate	Pencil	12.92 (12.6–13.3) *	0.57 (0.55–0.58)	1.13 (1.10–1.17)
Locking Plate	Weight	X	X	X
Locking Plate	Jar	X	X	X
Tension Band	Typing	2.11 (1.7–2.5)	0.09 (0.07–0.11)	0.18 (0.15–0.22)
Tension Band	Piano	5.87 (5.6–6.1)	0.26 (0.24–0.27)	0.51 (0.49–0.53)
Tension Band	Pencil	5.29 (5.0–5.6)	0.23 (0.22–0.24)	0.46 (0.44–0.49)
Tension Band	Weight	16.13 (15.0–17.3)	0.71 (0.66–0.76)	1.42 (1.32–1.52)
Tension Band	Jar	24.00 (22.0–26.0)	1.06 (0.97–1.15)	2.13 (1.94–2.31)

## Data Availability

The data presented in this study are available on request from the corresponding author.

## Abbreviations

The following abbreviations are used in this manuscript:

PIJ	Proximal interphalangeal joint
ADLd	Activities of daily living
IFS	Interfragmentary strain
DIJ	Distal interphalangeal joint

## Appendix A

**Table A1:** Iterations of force–angle correlations for different techniques in extension or flexion.

Technique	Extension	Flexion
Cerclage	angle = −1.4753 + 1.2478 × √force	angle = −0.0918 + 1.1450 × √force
Compression wire	angle = −1.4251 + 1.1484 × √force	angle = −3.7193 + 3.4213 × log(force)
Fixation plate	angle = −1.2935 + 0.3699 × force^2^	angle = −0.9852 + 2.077 × log(force)
Herbert screw	angle = −1.3818 + 1.1291 × √force	angle = −2.8308 + 1.8937 × √force
K-Wire	angle = −2.9143 + 1.9913 × √force	angle = −4.2867 + 3.9663 × log(force)
Locking plate	angle = 0.6116 + 0.0426 × force^2^	angle = −3.2307 + 2.5416 × log(force)
Tension band	angle = −3.5851 + 2.1517 × √force	angle = −2.5469 + 2.9747 × log(force)
